# Long read sequencing to reveal the full complexity of a plant transcriptome by targeting both standard and long workflows

**DOI:** 10.1186/s13007-023-01091-1

**Published:** 2023-10-21

**Authors:** Othman Al-Dossary, Agnelo Furtado, Ardashir KharabianMasouleh, Bader Alsubaie, Ibrahim Al-Mssallem, Robert J. Henry

**Affiliations:** 1 Queensland Alliance for Agriculture and Food Innovation, University of Queensland, Brisbane, 4072 Australia; 2 College of Agriculture and Food Sciences, King Faisal University, 36362 Al Hofuf, Saudi Arabia; 3 ARC Centre of Excellence for Plant Success in Nature and Agriculture, University of Queensland, Brisbane, 4072 Australia

**Keywords:** Jojoba, Transcriptome, Iso-Seq, Isoforms, *Simmondsia chinensis*, Gene splicing, Gene expression

## Abstract

**Background:**

Long read sequencing allows the analysis of full-length transcripts in plants without the challenges of reliable transcriptome assembly. Long read sequencing of transcripts from plant genomes has often utilized sized transcript libraries. However, the value of including libraries of differing sizes has not been established.

**Methods:**

A comprehensive transcriptome of the leaves of Jojoba (*Simmondsia chinensis*) was generated from two different PacBio library preparations: standard workflow (SW) and long workflow (LW).

**Results:**

The importance of using both transcript groups in the analysis was demonstrated by the high proportion of unique sequences (74.6%) that were not shared between the groups. A total of 37.8% longer transcripts were only detected in the long dataset. The completeness of the combined transcriptome was indicated by the presence of 98.7% of genes predicted in the jojoba male reference genome. The high coverage of the transcriptome was further confirmed by BUSCO analysis showing the presence of 96.9% of the genes from the core viridiplantae_odb10 lineage. The high-quality isoforms post Cd-Hit merged dataset of the two workflows had a total of 167,866 isoforms. Most of the transcript isoforms were protein-coding sequences (71.7%) containing open reading frames (ORFs) ≥ 100 amino acids (aa). Alternative splicing and intron retention were the basis of most transcript diversity when analysed at the whole genome level and by specific analysis of the apetala2 gene families.

**Conclusion:**

This suggests the need to specifically target the capture of longer transcripts to provide more comprehensive genome coverage in plant transcriptome analysis and reveal the high level of alternative splicing.

**Supplementary Information:**

The online version contains supplementary material available at 10.1186/s13007-023-01091-1.

## Background

The plant transcriptome comprehensively includes all the expressed genes in a plant cell or tissue. The construction of a reference transcriptome requires RNA sequencing to determine the identity of all the transcripts. RNA sequencing (RNA-seq) can be used to investigate the variation in expression of these transcripts in response to developmental or environmental events [1]. The use of short-read sequencing data has limitations to construct a complete reference transcriptome due to the potential occurrence of errors in assembly of the transcript isoforms [2]. The PacBio isoform sequencing (Iso-Seq) method can provide longer sequencing reads with uniform coverage and define the diversity of full-length transcripts without requiring assembly [3, 4]. Unlike RNA short-read sequencing, Iso-Seq allows for a better understanding of the transcriptome complexity including alternative splicing events (ASEs) [5, 6], non-coding RNAs (ncRNAs) [7], nonsense-mediated mRNA decay (NMD), and alternative polyadenylation [8]. Despite the higher costs of Iso-Seq technology, it enhanced the ability to explore plant gene expression and construct a complete reference transcriptome [9]. To date, Iso-Seq has been widely used in transcriptomic research and enhanced the field of the functional studies in several plant species. In sugarcane, a total of 107,598 unique transcript isoforms were generated using PacBio Iso-Seq and represented 71% of the predicted genes [1]. In arabica coffee (*Coffea arabica*), 95,995 unique transcript isoforms were detected to construct a comprehensive reference transcriptome [10]. In opium poppy (*Papaver somniferum*), Iso-Seq technology improved genome annotation and detected genome-wide alternative splicing events (ASEs). The construction of opium Iso-Seq transcriptome corrected 1007 misannotation, identified 10,473 novel genes, and 474,169 novel ASEs [11]. A total of 38,556 high-quality (HQ) isoforms post Cd-Hit transcript isoforms were generated to create a turfgrass (*Festuca brevipila*) reference transcriptome with transcript lengths that ranged between 58 and 11,487 bp [12]. To this date, no evidence of using the two workflow libraries (standard and long) was found in the literature.

The Iso-Seq construction pipeline starts with isolating high-quality RNA, followed by cDNA synthesis, size-partitioning, and amplification [13]. A standard SMRTbell™ library is prepared, loaded into a SMRT^®^ Cell, and placed in the instrument for sequencing [14]. The preferential sequencing of short transcripts in PacBio sequencing has been widely recognized. Transcripts have often been fractionated to allow separate size transcripts to be sequenced separately. Up to four fractions have been generated and combined, but due to the expense of sequencing multiple libraries, more recent studies often analyse a single standard library preparation. In this study we have investigated the consequences of using a standard library alone and the additional information provided by sequencing a library selected to be longer. While the use of the standard workflow alone can generate new isoforms with high accuracy, it may lead to the omission of valuable longer isoforms. In the current study, Iso-Seq technology was used to decipher the complexity of jojoba reference transcriptome. Jojoba is a common dioicous perennial water-stress tolerant shrub from the Sonoran Desert. The available genomic and transcriptomic database of jojoba is still limited. The growing interest in jojoba led the scientific community to construct the first plant reference genome of both sexes and reveal major differences in the sex chromosomes [15]. Recently, jojoba reference transcriptome was de novo assembled using Illumina short-read sequencing to investigate the seeds lipid biosynthetic pathway [16]. These two studies have enriched jojoba database, however a jojoba reference transcriptome based on PacBio long-read sequencing is still needed. The objective of this study was (i) to comprehensively sequence and functionally annotate a long-read reference transcriptome for jojoba; (ii) to compare jojoba long-read transcripts from the two different library preparations: standard and long; and (iii) to characterize alternatively spliced transcript isoforms. This work aimed to determine the significance of use of the standard and long datasets and establish an Iso-Seq reference transcriptome to allow detailed studies on the genetic basis of drought tolerance, sex determination, and the liquid-wax oil production in jojoba.

## Results and discussion

### Jojoba transcriptome from PacBio isoform sequencing

Poly(A)-RNA extracted from leaves of six jojoba plant samples was sequenced using a PacBio Sequel II instrument. This sequencing aimed to achieve high coverage of the jojoba transcriptome and produce an Iso-Seq reference transcriptome. Only CCSs (circular consensus sequences) derived from a minimum of seven passes of the insert were considered. A total of 5,402,043 CCS reads were generated from the long-read sequencing platform using two different Iso-Seq library workflows: the standard workflow (SW) with 2,751,112 reads, and the long workflow (LW) with 2,650,931 reads. The CCS reads from both workflows were combined to maximize transcript coverage in the Iso-Seq functional annotation process and eliminate potential redundancy. This is the first study to use the two workflows SW and LW to establish a high-quality transcriptome Iso-Seq reference that can include most of the isoforms with longer sequences. The combined data from the two datasets yielded 426,380 clustered isoforms, demonstrating that 74.6% of the sequences were unique to either the standard or long datasets. Subsequently, the merged dataset (reference Iso-Seq) was subjected to the Cd-Hit redundancy removal with 99% similarity to produce HQ isoforms post Cd-Hit and unique isoforms. Cd-Hit is a software application designed for clustering and comparing protein or nucleotide sequences, aiming to minimize sequence redundancy and enhance the efficiency of sequence analysis. The final HQ isoforms post Cd-Hit Iso-Seq reference had a total of 167,866 transcript isoforms. The total number of transcript isoforms represents genes found exclusively in leaf tissue. A higher count is anticipated when incorporating data from other plant tissues. The length of the total isoforms reads ranged from 56 to 9099 bp, with an average length of 2958 bp. The GC content was 41.1%, and the N50 was 3279 bp (Table 1). In a recent jojoba transcriptome study [16], Illumina short-read sequencing was used to de novo assembled a reference jojoba transcriptome for developing seeds. The constructed transcriptome had a total of 167,684 transcripts with an average length of 600 bp, N50 of 830 bp, and a GC content of 39.9%. The current study has provided a high-quality jojoba transcriptome with slightly higher number of isoforms (167,866) and greater average length of 2958 bp compared to only 600 bp. This difference between the two studies is expected due to the use of different short-read and long-read sequencers NovaSeq 6000 and PacBio, respectively in addition to the inclusion of both SW and LW datasets. In a different comparison to the turfgrass (*Festuca brevipila*) transcriptome, the combination of using both SW and LW datasets in the current study has led to a better transcriptome construction. The turfgrass transcriptome consisted of a total of 38,556 isoform transcripts, averaging 2147 bp in length, with an N50 of 2584 bp [12]. A majority of the transcripts (83.3%) exceeded a length of 1000 bp. In this study, the jojoba transcriptome exhibited a higher numbers of transcript isoforms, an increased average length, a higher N50 value, and a greater percentage, with 99.2% of transcripts exceeding a length of 1000 bp.

**Table 1:** Summary statistics of *S. chinensis* high-quality (HQ) isoforms and HQ isoforms post Cd-Hit datasets

	HQ Isoforms		HQ Isoforms post Cd-Hit		
	Standard	Long	Standard	Long	Merged
Number of sequences	262,206	216,879	106,568	103,904	167,866
Total length (Mb)	568	690	261	347	493
Longest sequence (bp)	8373	8991	8373	8991	9099
Shortest sequence (bp)	53	77	69	106	56
Mean sequence length (bp)	2166	3184	2451	3347	2939
Median sequence length (bp)	2094	3058	2365	3232	2873
Number of sequences > 10^3^ (bp)	231,231 (88.2%)	215,162 (99.2%)	99,680 (93.5%)	103,082 (99.2%)	161,419 (96.1%)
GC content (%)	42.2	41.6	41.4	41.0	41.1

The HQ isoforms and HQ isoforms post Cd-Hit datasets represent the standard workflow (SW) and long workflow (LW), in addition to the final transcriptome Iso-Seq reference dataset which includes the merged dataset of the HQ isoforms post Cd-Hit standard and long workflows (SW + LW)

**Fig. 1:**
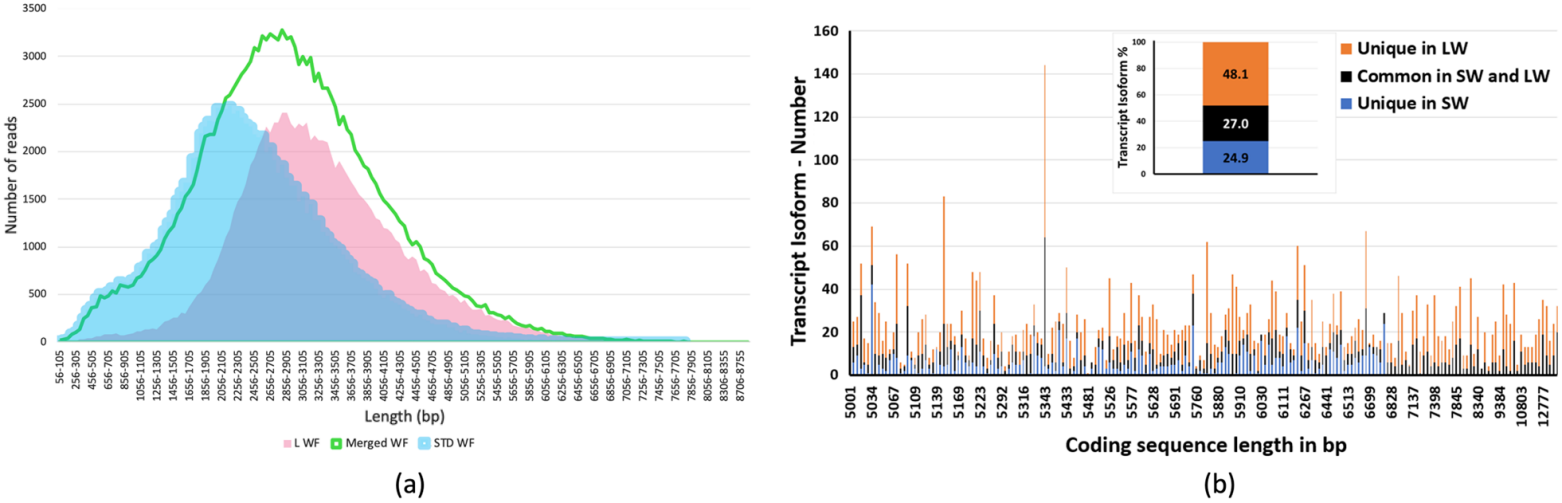
Comparison between isoforms transcripts obtained from standard and long datasets. **a** size distribution of transcript isoforms from three high-quality (HQ) isoforms posts Cd-Hit different datasets: standard (SW) (light blue), long (LW) (pink), and merged dataset (MW) (green) derived from jojoba (*Simmondsia chinensis*) transcriptome Iso-Seq reference. **b** distribution of unique isoforms transcripts from the long workflow across genes with CDS length of over five thousand base pairs. Out of a total of 41,539 full length CDS sequences [15], 201 CDS had length over 5000 bp and of these 196 had at least one mapped transcript isoform. For the 196 CDS sequences, a total of 4,969 common transcript isoforms between SW and LW were mapped with 2388 unique transcripts generated by the LW. Across all the isoforms, the average ratio of unique isoforms generated by LW/total isoforms by the SW generated isoforms was 1.03. Common transcript isoforms derived from SW and LW mapped to each of the 196 CDS are shown as blue bars and had a percentage of 24.9%, the unique transcripts isoforms derived from the SW are shown as black bars and had a percentage of 27.0%, while the unique transcripts isoforms derived from the LW and shown as orange bars had a percentage of 48.1%. Transcript isoforms shown here are Cd-Hit CCS sequences at 99% similarity. Mapping was undertaken using the “enable long-read spliced alignment” option in Minimap2 and executed via the CLC Genomics Workbench. This data contrasts with the previous report of Illumina short transcripts assembled to construct a jojoba reference transcriptome [16]

The two datasets, SW and LW, displayed some unique differences (Fig. 1a). In the HQ isoforms dataset, the SW length ranged between 53 to 8373 bp, with an average length of 2166, whereas the LW had a length range of 77 to 8991 bp and a higher average length of 3184 bp. The SW dataset had a GC content of 41.6% compared to the LW dataset with 42.2%. Comparison of the three HQ isoforms post Cd-Hit datasets (only CCS reads from standard library, only CCS reads from long library, and merged CCS reads) confirmed the higher quality of the merged workflow (Table 1). The LW dataset produced fewer (103,904 isoforms) but longer transcript isoforms (average length: 3347 bp) compared to the SW dataset which generated more (106,568 isoforms) but smaller transcript isoforms (average length: 2451 bp). However, the merged dataset had a higher number of long isoforms (167,866, average length: 2939 bp). The substantial number of isoforms in the merged dataset suggested a comprehensive coverage of more and longer isoforms. This is due to the unique and long isoforms contributed only by the LW dataset as shown in the contrasting peaks of both SW and LW in Fig. 1a.

### Characterisation of jojoba large gene-related isoforms reveals distinct contribution from the long workflow (LW) dataset

**Fig. 2:**
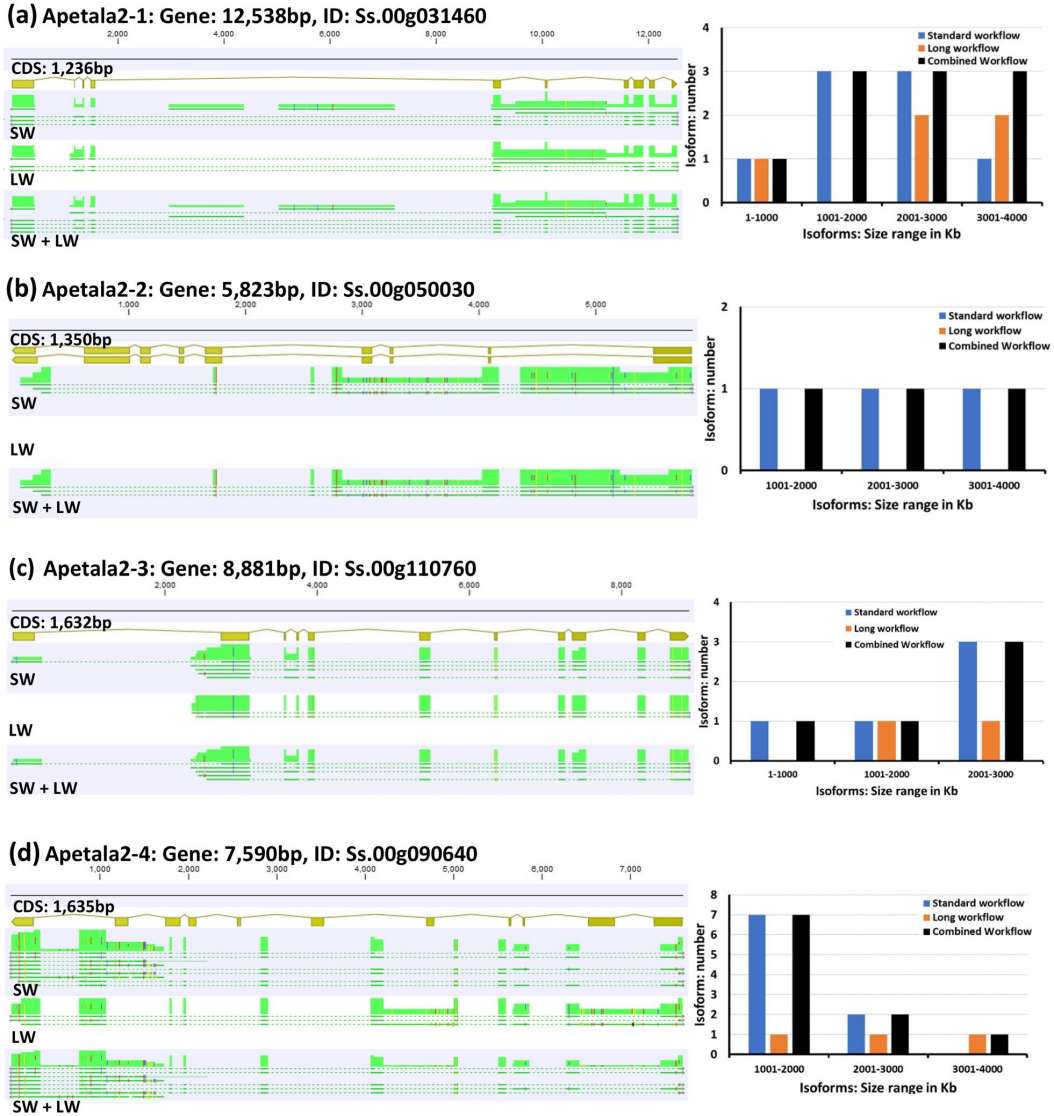
Comparison of PacBio Iso-Seq standard and long workflows derived transcripts isoform sequences using the apetala genes with annotated coding sequences under 5000 bp. PacBio isoforms were generated using the standard workflow (SW) and the long workflow (LW). Circular consensus read redundancy in both datasets was removed using high quality high-quality (HQ) isoforms post Cd-Hit at 0.99% before combining to then generate SW + LW. Unique LW-derived transcript isoform/s are present if the black bar is higher than the blue bar in any specific isoform size range or if absence of a blue bar and presence of an orange bar. Number and length distribution of transcript isoforms derived from the SW, LW and SW + LF mapped to four apetala genes are shown. The HQ isoforms post Cd-Hit data indicates similar sequence in both workflows Transcript isoforms except for one unique isoform for Apetala2-1 gene at the size range 3001 bp to 4000 bp. Mapping was undertaken using the “enable long-read spliced alignment” option in Minimap2 and executed via the CLC Genomics Workbench

The apetala2 *(apt2)* (Accession#: HQ637468) gene belongs to a large transcription factor family and is responsible for the determination of the plant floral structure and meristem. Apetala2 genes were used to explore the diversity of the jojoba transcriptome, given that Apetala2 is a sex-related gene located on the jojoba male Y chromosome and has been proposed to play a significant role in plant sex determination [15]. Based on the recently published jojoba reference genome, four apetala2 genes (apetala2-1: Ss.00g340210, apetala2-2: Ss.00g212550, apetala2-3: Ss.00g340210, apetala2-4: Ss.00g048870) were located on chromosomes 9, 4, 9, and 2, respectively [15]. The apetala2-1 and apetala2-3 were male-specific, while the other two genes, apetala2-2 and apetala2-4, were present elsewhere in the genome. The four genes had a length that ranged between 5800 bp and 12,500 bp, with an exon number range of 9–11 exons (Table 2). The jojoba HQ isoforms post Cd-Hit Iso-Seq reference was aligned to the four apetala2 gene sequences (Fig. 2). The four apetala2 genes (*ap2-1*: Ss.00g340210, *apt2-2*: Ss.00g212550, *apt2-3*: Ss.00g340210, and *apt2-4*: Ss.00g048870) had 10, 3, 5, and 10 isoforms, respectively (Table 2). The highest average coverage was identified for the *apt2-2* isoforms with 43%, where the lowest was for the isoforms mapped to the *apt2-1* with only 18% (Table 2, and Additional file 1: Table S7). This finding suggests that a substantial number of unique APETALA2 isoforms were associated with *apt2-1* and *apt2-4*. (Fig. 4c). Differences in the structural annotation of these four apetala genes could be used to study the expression of these genes and their role in the biology of the plant.

**Table 2:** Coding potential and SQANTI3 analyses of four Apetala2 genes isoforms of *S. chinensis* reference genome

Genes Details				Isoforms Details								
Gene	Size (bp)	Exon count	Average coverage%	Name	Size (bp)	Coding potential	Chr	Structural category	Sub-category	All-canonical	Standard/long	
Apetala2-1	12,538	10	18	ISO/171107	2824	complete	chr9	ISM	Intron retention	canonical	Yes	
				ISO/105509	3316	complete	chr9	NIC	Intron retention	canonical		Yes
				ISO/412095	643	3’ partial	chr9	NIC	mono-exon	NA		Yes
				ISO/327430	1787	complete	chr9	ISM	3’fragment	canonical	Yes	
				ISO/315531	1880	complete	chr9	NIC	Intron retention	canonical	Yes	
				ISO/222064	2503	complete	chr9	NNIC	Intron retention	canonical		Yes
				ISO/366229	1420	Non	chr9	Intergenic	mono-exon	NA		Yes
				ISO/270607	2192	Non	chr9	Intergenic	mono-exon	NA	Yes	
				ISO/100081	3363	complete	chr9	ISM	Intron retention	canonical		Yes
				ISO/111394	3253	complete	chr9	NIC	Intron retention	canonical	Yes	
Apetala2-2	5823	9	43	ISO/303053	1970	complete	chr4	NIC	Intron retention	canonical	Yes	
				ISO/257514	2274	5’ partial	chr4	ISM	Intron retention	canonical	Yes	
				ISO/105872	3312	5’ partial	chr4	NIC	Intron retention	canonical	Yes	
Apetala2-3	8881	11	21	ISO/265232	2225	complete	chr9	NNIC	At least one novel splicesite	canonical	Yes	
				ISO/264460	2218	complete	chr9	ISM	3’ fragment	canonical	Yes	
				ISO/253270	2302	complete	chr9	NIC	Intron retention	canonical	Yes	
				ISO/409273	705	3’ partial	chr9	NIC	mono-exon	NA		Yes
				ISO/319944	1834	complete	chr9	NNIC	Intron retention	noncanonical	Yes	
Apetala2-4	7590	11	25	ISO/361458	1476	complete	chr2	ISM	5’ fragment	canonical	Yes	
				ISO/358337	1508	complete	chr2	NNIC	At least one novel splicesite	noncanonical		Yes
				ISO/382288	1207	complete	chr2	NNIC	At least one novel splicesite	noncanonical		Yes
				ISO/303247	1969	complete	chr2	ISM	5’ fragment	canonical	Yes	
				ISO/295283	2026	complete	chr2	NNIC	At least one novel splicesite	canonical	Yes	
				ISO/297282	1976	complete	chr2	FSM	At least one novel splicesite	canonical	Yes	
				ISO/293887	2035	complete	chr2	NNIC	At least one novel splice site	noncanonical	Yes	
				ISO/320398	1844	complete	chr2	Genic	mono-exon	NA	Yes	
				ISO/99492	3371	5’ partial	chr2	NNIC	Intron retention	canonical		Yes
				ISO/321893	1832	5’ partial	chr2	FSM	Alternative 3/5 ends	canonical	Yes	

Coding potential analysis categorize the isoforms into the following categories: complete, 5’ prime (5’ prime), 3’ prime (3’ prime), and no coding potential. SQANTI3 analysis result includes the following information: chromosome number (Chr), structural category, sub-category, and all-canonical. The structural category is divided into intergenic, genic, full-splice match (FSM) incomplete full-splice match (ISM), novel not in catalog (NNIC), and novel in catalog (NIC) transcripts. The sub-category is classified into intron retention (IR), mono-exon, 5’ prime fragment, 3’ prime fragment, Alternative 3end 5end, and at least one novel splice site. Every isoform size and dataset type (standard or long) were determined

**Fig. 3:**
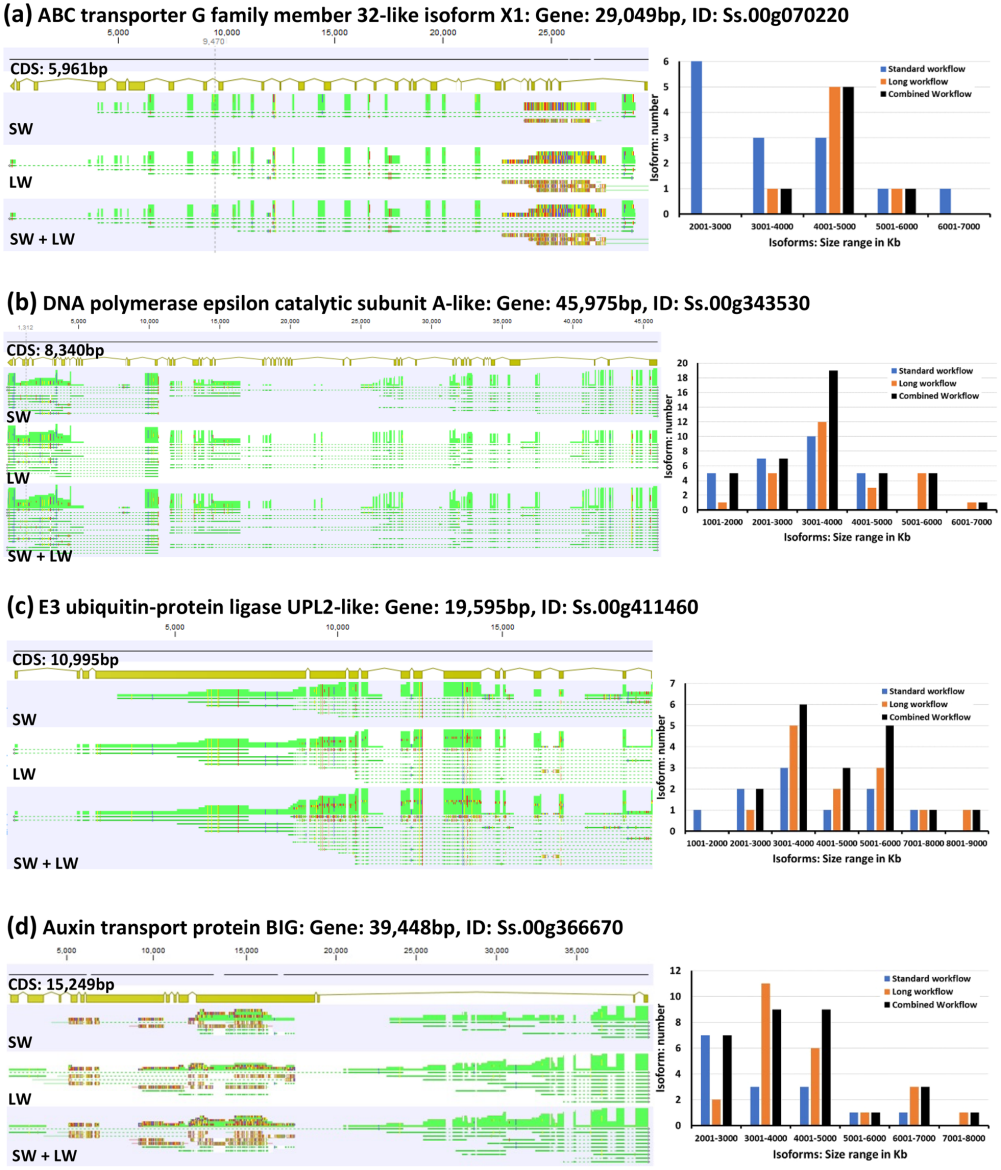
Comparison of PacBio Iso-Seq standard and long workflow derived transcripts isoform sequences using four genes with annotated coding sequences above 5,000 bp. PacBio isoforms were generated using the standard workflow (SW) and the long workflow (LW). Circular consensus read redundancy in both datasets was removed using high-quality (HQ) isoforms post Cd-Hit at 0.99% before combining to then generate SW + LW. Unique LW-derived transcript isoform/s are present if the black bar is higher than the blue bar in any specific isoform size range or if absence of a blue bar and presence of an orange bar. Number and length distribution of transcript isoforms derived from the SW, LW and SW + LF mapped to four genes are shown. LW-derived unique isoforms were generated for all four genes. Mapping was undertaken using the “enable long-read spliced alignment” option in Minimap2 and executed via the CLC Genomics Workbench

**Fig. 4:**
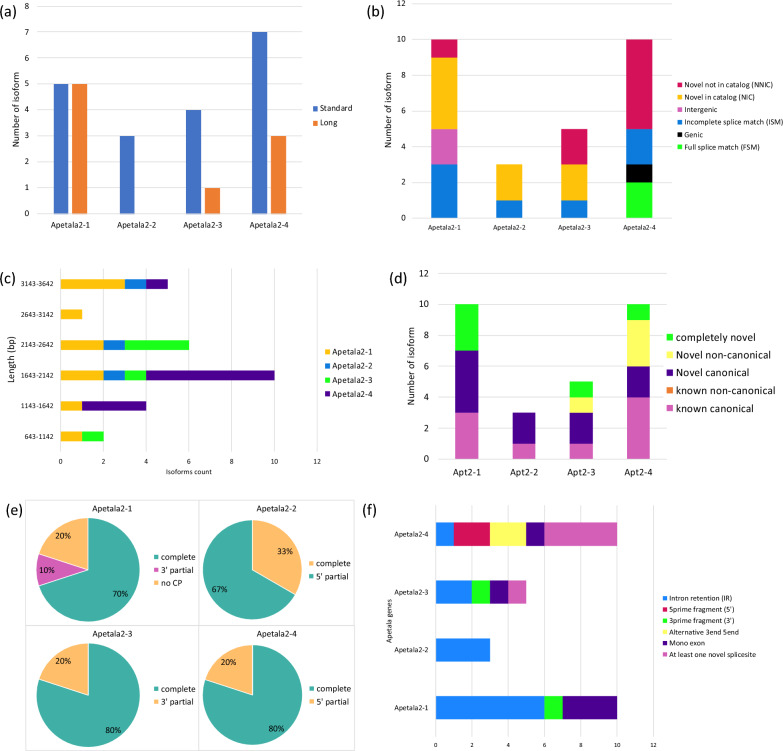
The SQANTI3 and coding potential analyses for the four Apetala2 genes obtained from jojoba (*Simmondsia chinensis*) reference genome. **a** isoforms count frequency per library size type of standard and long. **b** distribution of SQANTI3 structural classification including incomplete full-splice match (ISM), novel in catalog (NIC), novel not in catalog (NNIC), intergenic, and genic isoforms. **c** isoforms count frequency for the four Apetala2 genes by their length in base pair (bp). **d** distribution of SQANTI3 canonical and non-canonical isoforms for the known and novel genes for all the four apetala genes plus the number of the completely novel isoforms with unknown sequence identification. **e** the coding potential for the four Apetala2 genes isoforms obtained from the jojoba (*Simmondsia chinensis*) reference genome and Iso-Seq reference. The coding potential including the four categories: complete, 5’ potential, 3’ potential, and noncoding sequences. **f** sub-category classification for the four Apetala2 genes including intron retention (IR), 5 prime fragment, 3 prime fragment, alternative 3 end 5 end, and at least one novel splice site

Multiple transcript isoforms from both the SW and LW datasets mapped to each of the APETALA genes, revealing several spliced variants in accordance with the CDS annotations (Fig. 2). Several LW-isoforms were identified in three apetala genes. The *apt2-1* and *ap2-4* genes had the largest number of LW-isoforms of five and three isoforms, respectively, whereas *apt2-3* had only one LW-isoform (Fig. 4a). All the *apt2-2* related isoforms belonged to the SW dataset (Table 2). The LW dataset was constructed to capture longer isoforms that might not have been detected by the SW, resulting in the generation of unique isoform transcripts exclusively from the LW dataset. This was especially the case for transcripts coding sequences longer than 5kb. In the case of CDS sequences ranging form 1kb to 3kb and 3kb to 5kb, unique isoform transcripts were identified from the LW (Additional file 1: Figures S1 and S2) with an average ratio of LW unique transcript isoforms to those from the SW of 0.65 and 1.0, respectively (Additional file 2: Tables S1 and S2). The percentage of the isoform transcripts contributed by the LW in the CDS sequences ranging from 1 to 3 kb was 37.1%, whereas the unique SW isoforms transcripts accounted for 36.3% (Additional file 2: Tables S1). In the CDS sequences ranging from 3 to 5 kb, the LW accounted for 47.9% of unique isoform transcripts, whereas the unique SW isoform transcripts constituted 26.9% (Additional file 3: Tables S2). In the CDS sequences above 5kb, unique isoform transcripts were identified from the LW (Fig. 1b) with an average ratio of LW unique transcript isoforms to those from the SW of 1.03 (Additional file 4: Table S3). The LW dataset has contributed with unique transcript isoforms of 48.1% comparing to 24.9% of the SW dataset, and 27.0% from the common transcript isoforms between the two datasets (SW and LW). Mapping patterns and isoform length distribution of the apetala2 genes with CDS length under 5kb identified no unique LW derived transcript isoforms for the apetala genes except for one isoform for the apetala2-1 gene (Fig. 2a). However, mapping patterns and isoform length distribution of five other genes with CDSs above 5kb, indicated that shorter transcripts spliced variants were generated in the SW and longer isoforms were predominantly generated from the LW (Fig. 3). The mapping patterns of the transcript isoforms matched the CDS annotation only for the apetala2-3 and E3 ubiquitin-protein ligase genes but not for the other genes (Fig. 2 and 3). This indicates that the common incomplete reference annotation issue for the non-model plants can be enriched using the Iso-Seq transcriptome of the combined datasets of SW and LW [17, 18]. The unique Iso-Seq transcripts contributed especially by LW dataset would serve as a valuable resource for future reference annotation improvement using tools such as tappAS [19] and IsoformSwitchAnalyzeR [20].

Each group of isoforms was subjected to both coding potential and SQANTI3 to understand their functionality and to identify any variation. Of the ten isoforms mapped to the *apt2-1* (Ss.00g340210), seven had complete coding potential, one was a 3’ prime transcript, and two had no coding potential. Two of the *apt2-2* gene (Ss.00g212550) isoforms were 5’ prime, and only one isoform had no coding sequence. Moreover, the third *apt2* gene (Ss.00g340210) had five isoforms, where four of them were complete and had 3’ prime sequences. Of the ten isoforms mapped to the apt2-4 (Ss.00g048870), eight were complete coding sequences and two were 5’ prime. Overall, 90% of the apetala2 genes-related isoforms had coding potential. The high percentage of the coding isoforms among the apetala2 genes indicates their functionality in jojoba sex determination (Fig. 4d).

This highly functional group of apetala2-related isoforms were also used in SQANTI3 analysis to detect their splicing pattern and to understand their distinctions. The SQANTI3 tool was created for the comprehensive characterization and curation of long-read transcriptomic data. It achieves this by leveraging transcript data, genome annotation, and optionally quantification data to produce an extensive collection of transcripts and junction descriptors, as visualized in multiple diagnostic plots. The SQANTI3 approach is the ideal tool to ensure capturing the biological condition of the transcripts’ sequences. The structural classification of SQANTI3 are classified into eight groups: isoform transcripts with full splice junctions match to the reference genes (FSM), isoform transcripts with incomplete splice match to the reference genes (ISM), novel isoform transcripts with new combination for previously identified donor or acceptor sites named as novel in (NIC), isoform transcripts contain at least one novel donor or acceptor site designated as novel not in catalog (NNIC), antisense if an isoform with poly(A) overlap the complementary strand of an annotated gene, fusion where isoform transcripts integrate two annotated reference gene loci, genic are isoform transcripts which partially contain exon and intron/intergenic overlap of already annotated reference gene, and isoform transcripts in novel genes which lying outside the annotated gene boundaries known as intergenic. In the structural classification of SQANTI3, the ISM category was present in all the four apetala2-related isoforms with the *apt2-1* having the highest number of ISM isoforms (Fig. 4b). The NNIC and NIC structural categories were detected in three different apetala2 genes. The NNIC isoforms were found in the *apt2-1*, *apt2-3*, and *apt2-4* with the *apt2-4* being the highest, where the NIC isoforms were identified in the *apt2-1*, *apt2-2*, and *apt2-3* with the *apt2-1* having the highest number of NIC isoforms. Furthermore, the FSM and the genic categories were determined only in the *apt2-4* with two and one isoforms, respectively, where two Intergenic isoforms were found exclusively in the *apt2-1*-realted isoforms. This result suggests that the two structural classifications, NNIC and NIC, consistently included the highest number of isoforms among the SQANTI3 structural classifications, as evidenced by the novel apetala2 isoform sequences in the jojoba Iso-Seq reference. The presence of a large number of novel isoforms among apetala2 genes aligns with the overall SQANTI3 structural classification for the jojoba Iso-Seq reference and highlights a substantial, previously undiscovered transcriptional diversity.

### Identification of alternative splicing events (ASEs)

The isoform sequencing contributes to the characterization of alternative splicing events (ASEs) [7]. In plants, ASEs facilitate the regulation of around 70% of multi-exon genes [21]. ASEs of multi-exon genes, such as apetala2, help to create a complex functional transcriptome which can regulate different plant biological processes [22, 23]. The ASEs of the four apetala2 genes were determined using SQANTI3. The most frequent type of ASEs among the four apetala2 genes was intron retention (IR). The *apt2-1* gene had the highest number of IR ASEs, followed by *apt2-2*, *apt2-3*, and *apt2-4*. The high number of IR events in jojoba apetala2 genes agrees with previous findings which confirm the predominance of IR ASE in plants [2, 21, 24]. The ASE within at least one novel splice site contributed to isoforms across the two apetala2 genes of *apt2-3* and *apt2-4* with 1 and 5 isoforms, respectively. Other ASEs were also present in the *apt2-1*, *apt2-3*, and *apt2-4* such as, 3’ and 5’ prime fragments and alternative 3’ end and 5’ end (Fig. 4f). Further, the canonical and non-canonical splicing events are highly conservative and expressed modifications which are catalysed by spliceosome [25]. In this study, the splicing events of canonical and non-canonical isoforms were determined for the four apetala genes regarding the known versus novel isoforms transcripts. The *apt2-4* had the highest number of known canonical and novel non-canonical isoforms with four and three isoforms, respectively. The maximum number of novel canonical isoforms was found in *apt2-1* [4] (Fig. 4d).

### Isoform classification with SQANTI3

**Fig. 5:**
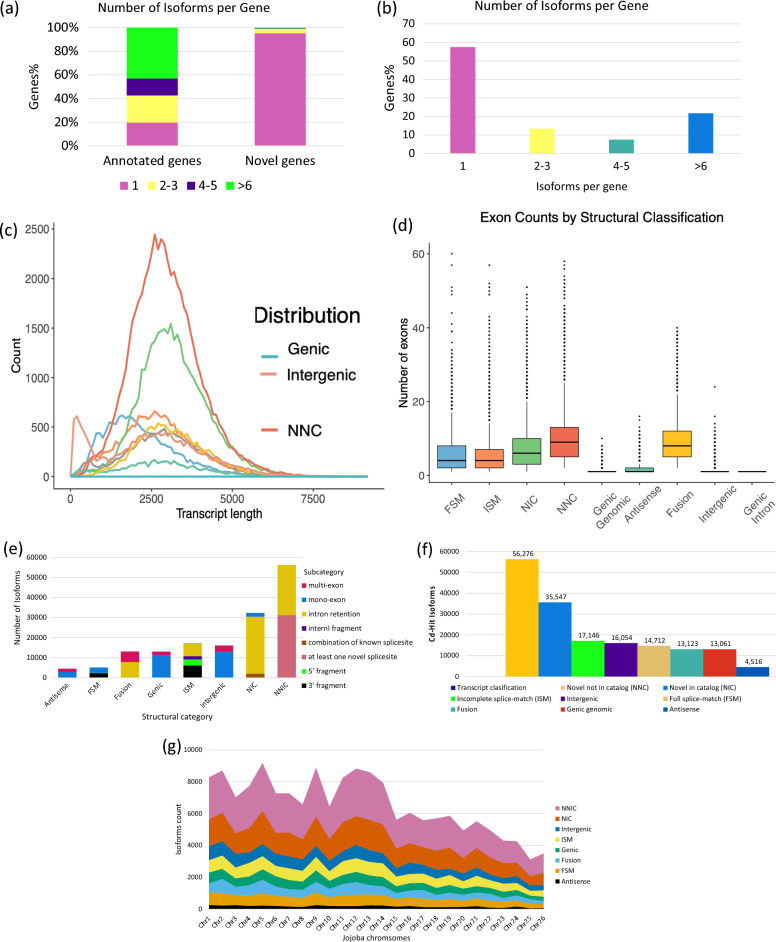
The structural classification of jojoba (*Simmondsia chinensis*) Iso-Seq reference categorised by SQANTI3. **a** number of isoforms per both gene types: annotated and novel genes. **b** number of isoforms per gene. **c** structural classification frequency by transcript length in base pairs. **d** boxplot of structural classification by exon number. **e** isoforms count categorised by structural classification and subcategory classification. **f** distribution of SQANTI3 structural classification. **g** structural classification distribution aligned to jojoba reference genome chromosomes for (1) novel not in catalog, (NNIC), (2) novel in catalog (NIC), (3) intergenic, (4) incomplete full-splice match (ISM), (5) genic, (6) fusion, (7) full-splice match (FSM), and (8) antisense transcripts

SQANTI3 was utilized to correct, filter, and classify the jojoba isoforms based on the annotation of the jojoba male reference genome, employing various quality control metrics. The jojoba reference genome comprises a total of 37,680 genes, of which 57.4% correspond to one isoform, 20.9% have 2–3 isoforms, and 21.7% with more than six isoforms (Fig. 5b). Of 37,680, 18,870 genes were annotated and 18,810 were novel. Most the annotated genes have more than six isoform (42.9%), whereas 95.3% of novel genes had only one isoform (Fig. 5a). The structural classification of SQANTI3 classified jojoba Iso-Seq reference into eight groups: FSM, ISM, NIC, NNIC, antisense, fusion, genic, and intergenic. The majority of the jojoba isoforms were in the NNIC group (33.0%) followed by the NIC group (20.9%), and few of the isoforms were genic genomic (7.7%) and antisense (2.7%) (Fig. 5f). The total of the NNIC and the NIC isoforms represented more than half (53.9%) of the total jojoba isoforms. The result of the structural classification and exon distribution comparison showed that the two groups of antisense and genic genomic had the lowest exon number (Fig. 5d). Additionally, the total isoforms of 37,680 were also subclassified into the following subcategories: 5’ fragment (3020), 3’ fragment (6106), internal fragment (1666) and IR (67,682). The IR was found in the following subcategories: fusion, ISM, NIC and NNIC structural classifications, where multi-exon isoforms transcripts were present in the antisense, fusion, genic, and intergenic (Fig. 5e). The higher number of IR agrees with that found in Arabidopsis, which was estimated to be more than 30% [26]. There was no difference found in the structural classifications based on isoforms length distribution (Fig. 5c). The distribution of structural classification was aligned to the jojoba genome. The highest number of antisense, genic, and FSM isoform transcripts were determined to be from chromosome 1 and 9, whereas chromosomes 25, 26, and 24 had the lowest number of isoform transcripts (Fig. 5g). This indicates a higher ASEs in chromosome Y (chr9) which confirms its important role in the jojoba genome. The low number of genes showing ASEs in the following chromosomes chr24, chr25, and chr26 may be due to them being the smallest chromosomes (18.2, 17.9, 17.4 Mb) in the recently published jojoba genome [15].

### Transcriptome completeness

The completeness of the jojoba transcriptome libraries SW and LW was assessed by aligning them to the complete set of 37,680 jojoba genes, resulting in a high match percentage of 98.7% that indicates the comprehensiveness of the jojoba transcriptome. The Benchmarking Universal Single-Copy Orthologs (BUSCO) is an assessment tool that quantitatively evaluates the completeness of genomic data, gene sets, and transcriptomes by analysing gene content. BUSCO categorizes gene content into four groups: complete, duplicated, fragmented, and missing genes, utilizing recent data from a wide range of species. BUSCO was used to determine the completeness of the jojoba transcriptome libraries. The comparison of the complete genes percentage between the two workflows showed that the LW had a lower percentage of complete genes (375, 88.2%) compared to 409 (96.2%) for the SW (Additional file 4: Table S3). Merging the two workflow datasets produced a reference Iso-Seq with a higher percentage of complete BUSCO core genes (96.9%, 412), confirming the completeness of the transcriptome. BUSCO analysis with completeness scores of 96.2% for the SW and 88.2% for the LW involving enriched long transcript, increased slightly to 96.9% for the merged dataset. The marginal increase in BUSCO scores contributed by the LW and the presence of a large number of unique transcripts identified in the LW (Fig. 1b, and Additional file 1: Figure S2) indicates the presence of unique spliced isoforms and/or unique transcripts in the LW not represented in the Core Gene dataset used for the BUSCO analysis.

### Functional annotation of the reference transcriptome

Functional annotation was conducted based on the basic local alignment search tool (BLAST) with default settings to subject all the 167,866 transcript isoforms to different databases to investigate their function. A total of 115,176 of the transcript isoforms were assigned to gene ontologies (GOs). Out of the total transcripts, 21,549 corresponded to the InterProScan (IPS) database, while 17,208 were mapped to the non-redundent plant protein sequences (NR) database. Only 13,933 of the isoforms did not find any matches, suggesting that they may represent novel transcripts in the jojoba transcriptome. Of the 88,016 isoforms related to the IPS GOs, Phosphate-loop containing nucleoside triphosphate hydrolase (P-loop NTH) (IPR027417, 7597 matches) received most of the hits among the IPS families followed by the protein kinase-like domain superfamily (IPR011009, 6928 matches), and tetratricopeptide-like helical domain superfamily (IPR011990, 3636 matches) (Additional file 1: Figure S4). The P-loop NTH superfamily protein is a ubiquitous domain for many nucleotide-binding proteins [27]. The P-loop NTH superfamily proteins consist of 41 distinctive families of P-loop kinases or derived domains [28]. The P-loop NTH enzymes are well known catalyst of the hydrolysis of the phosphate bond of nucleoside triphosphate (*NTP*) [29]. The energy of the hydrolysis reaction is usually used to prompt configurational alterations in other molecules, which represents the foundation of the P-loop NTH biological functions [28]. In a study of the transcriptome of the Arabidopsis mediator23 (MED23) mutant, two P-loop NTH were found to be among 40 co-expressed genes associated with embryo, floral, or meristem development. The second largest superfamily was the protein kinase-like domain, which plays a role in the phosphor-transfer reaction [30]. In the past two decades, plant protein kinases were found to be involved in many signalling networks, such as pathogens detection, light perception, plant hormones, and different environmental stimuli [31–33]. In a transgenic study of Arabidopsis, the over expression of a calcium-dependent protein kinase (CDPK) was found to enhance the plant’s tolerance to drought stress [34]. The tetratricopeptide-like helical domain was the third largest superfamily (*TPH*) in the jojoba Iso-Seq transcriptome. The *TPH* proteins are involved in intermediate protein–protein interactions and synthesis of multiprotein complexes [35]. These proteins are involved in various biological processes, such as cell cycle division, transcriptional regulation, protein transportation, and protein folding [36]. The genes encoding for the *TPH* proteins were found to be highly expressed in the endosperm and involved in the production of protein and starch in both wheat and yam [37].

### Comparative Iso-Seq analysis of jojoba with closely related species

The functional annotation of jojoba Iso-Seq reference transcriptome data revealed a close relationship between jojoba and some other plant species based on the origin of the BLAST hits sequences. The highest number of BLAST hits, 163,144 matches, was retrieved from quinoa (*Chenopodium quinoa*), followed by beetroot (*Beta vulgaris subsp. vulgaris*) 110,709 matches, spinach (*Spinacia oleracea*) 110,552 matches, grapevine (*Vitis vinifera*) 92,230 matches, and riverbank grape (*Vitis riparia*) 46,250 matches. The first three plant species, quinoa, beetroot, and spinach were found to share the same order, *Caryophyllales,* with jojoba [38]. This finding was supported by the two jojoba genome publications [15, 39]. The determination of closely related species to jojoba will aid in explaining the plant's unique evolutionary history.

### KEGG pathway analysis

KEGG metabolic pathway analysis provides important evidence about the functionality of transcript isoforms [40]. A total of 350,208 transcript isoforms (82.1%) matched 323 KEGG pathway annotations. The spliceosome (ko03040) pathway had the highest number of transcript isoforms [6200 sequences] followed by starch and sucrose metabolism (ko00500, 5638 sequences), and biosynthesis of cofactors (ko01240, 5638 sequences) (Additional file 1: Figure S6).

Spliceosome is a ribonucleoprotein complex which activates the transition of RNA from pre-RNA to mature mRNA and it contribute to several RNA processes, such as alternative splicing (AS), transcription, translation, and localisation [41]. In an AS event, regulatory factors positioned within introns and exons will help to create different combination of exons and eventually generates multiple different mRNAs from a single gene [42]. In Arabidopsis, it is estimated that nearly 61% of intron-containing genes go through alternative splicing events [43]. The creation of AS transcripts boosts proteomic diversity, affects all aspects of plant development, and regulates many physiological processes linked to environmental stimulus [24, 42]. Genes which undergo AS were shown to have a higher evolutionary rate, and may be linked to better acclimation to environmental stressors [44]. Several transcriptomic studies have shown that stress-related ASEs are found in regulatory genes, such as transcription factors, protein kinases, and splicing factors [43, 45]. In a systematic transcriptomic comparison study of salt tolerance of contrasting rice genotypes, ASEs were either highly (*bZIP*) or exclusively expressed (*WRKY30*) in the tolerant genotype [46]. Consequently, the considerable number of ASEs in jojoba may be associated with ability to survive in a harsh desert environment.

The starch and sucrose metabolism pathway had the second-highest number of isoforms in jojoba. It includes many genes involved in osmoregulation and supplied energy to the stressed plants by generating soluble sugar and protein [47]. The biosynthesis of the cofactor’s pathway was the third-highest pathway in isoform number. In a study done by [48] on a drought-tolerant genotype of banana “Saba”, the biosynthesis of cofactors pathway was among the top KEGG pathways responding to drought stress [48]. Several other water-stress related KEGG pathways were detected, such as protein processing in the endoplasmic reticulum, plant-pathogen interaction, RNA transport, endocytosis, oxidative phosphorylation, and carbon fixation in photosynthetic organisms [49–53].

### Water-stress related transcription factors

A total of 7357 isoforms (4.4%), representing regulatory factors, were detected in the jojoba reference transcriptome. The top three regulatory factors with the highest number of isoforms belonged to the following families: mediator of RNA polymerase II transcription (*Med*) (260 isoforms), *WRKY* (252 isoforms), and *bHLH* (246 isoforms). The *Med* transcription factor family received the highest number of isoforms (260), which plays an essential role in the transcriptional machinery in eukaryotes, including plants. In Arabidopsis, the co-regulator (Med) was found in a stress-responsive transcriptional regulation network of 1500 transcription factor alongside RNA polymerase II (pol II) and general transcription factors (*GTFs)* [54, 55]. Many of the *Med*s have been involved in stress-responsive signaling pathways such as *Med9*, *Med16*, *Med18*, and *Med25* [56]. In an Arabidopsis water-stress experiment, *Med25* was found to be a co-repressor of *DREB2A* for both water-stress tolerance and flowering. The interruption of the *DREB2A-Med25* repression effect led to an elevated water-stress tolerance and early flowering [57]. The *WRKYs,* which has the second highest number of isoforms, plays an essential role in the plant response to water stress. The *WRKY* transcription factor is classified based on protein structural features into three groups (*WRKY- I, II,* and *III*) [58]. The *WRKYs* are stimulated by many abiotic stresses such as, water stress [59]. For instance, the overexpression of *WRKY33* in a transGenic Arabidopsis was found to improve its drought tolerance by engaging the plant *ABA* signaling during the stress [60]. Furthermore, the *bHLH* different stress-responsive subfamilies such as *IAA*-leucine resistant3 (*ILR3*), *IBH*, and inducer of *CBF* expression (*ICE*) were found to be involved in *ABA*-mediated stress tolerance as well as the interaction between jasmonic acid (*JA*) and *ABA* in response to stress [61, 62].

### Prediction of potential coding regions

The potential coding regions in the isoform sequences were investigated using Coding Potential Assessment Tool (CPAT) in OmicsBox to obtain open read frames (ORF). The CPAT utilises logistic regression to differentiate between protein-coding and non-coding transcripts. Of the 167,866 isoforms, 118,242 isoforms were detected to have 204,457 ORF sequences, representing 70.4% of the total reference transcriptome. Only 47,505 isoforms remained with no ORFs sequences. Of the total ORF sequences, 149,608 (73.2%) sequences had complete coding potential regions, 50,416 (24.7%) sequences had 5’ prime ORF sequences, 4361 (2.1%) sequences had 3’ prime ORF sequences, and 72 (< 1%) sequences had internal ORF sequences. The 5’ and 3’ prime ORFs represented sequences that lack the start and stop codon and possibly part of the N and C terminus, respectively (Additional file 1: Figure S7). The high proportion of complete coding transcripts (73.2%) comparing to only 58.1% from the recently published jojoba transcriptome indicates a comprehensive coverage of the jojoba transcripts in the present study [16]. Further, we also inspected the coding potential of isoforms with and without BLAST matches. Out of the 153,933 isoforms with BLAST matches and 13,933 without BLAST matches, 151,571 (98.5%) and 6413 (46.0%) were identified to have potential coding regions, respectively. This suggests the identification of a large number of functional novel jojoba isoform transcripts in the current transcriptome Iso-Seq reference transcriptome.

### Coding potential of jojoba novel isoforms

Out of the 167,866 isoforms in the jojoba Iso-Seq transcriptome, 8.3% [13] isoforms were identified to be novel. The novel isoforms sequences length ranges from 56 to 7587 bp. These novel isoforms showed no hit to any of the four major databases: the non-redundant (NR) plant proteins, the nucleotide collection (NT) database, InterProScan, gene ontology (GO). The potential coding regions in the isoform sequences were explored using CPAT in OmicsBox to obtain ORF, which showed their coding potential by sharing homology with the Pfam protein database. Of the 13,933 total novel isoforms in jojoba transcriptome Iso-Seq, 9969 represented ORFs sequences. Out of the total ORF sequences (9969), 7248 (72.7%) sequences exhibited complete coding potential regions, 2455 (24.6%) sequences had 5’ prime ORF sequences, 242 (2.4%) sequences had 3’ prime ORF sequences, and 24 (< 1%) sequences had internal ORF sequences (Additional file 1: Figure S8). In this study, the isoforms with coding potential are more likely to be novel genes that have not been annotated yet.

## Conclusions

The necessity of capturing the genome's diversity prompted the construction of a jojoba reference transcriptome, utilizing both standard and large insert datasets. Additionally, most genes in the jojoba genome were in the resulting dataset derived from leaf tissues alone. Moreover, transcript diversity was attributed to intron retention and alternative splicing. Notably, genes involved in the regulation of transcript splicing were abundant in the transcriptome, possibly due to the importance of splicing in the survival of jojoba in extreme environments. As a result, the findings of the present study will provide a rich basis for further jojoba research, particularly on water stress, sex determination, and seed wax content. Furthermore, it was crucial to target the specific sequencing of long transcripts to ensure their inclusion in the transcriptome, as long transcripts are often poorly represented in long-read transcriptomes produced for many plants.

## Materials and methods

### Plant materials and growth facility

Mature jojoba seeds from Saudi industrial varieties were obtained from King Faisal University (KFU) Al-Hofuf, Saudi Arabia (25° 16′15.1 "N 49° 42′42.6 "E). Jojoba seeds were cleaned, sterilized, and germinated. The jojoba seeds were grown for 3 months in a glasshouse of the University of Queensland (UQ) St Lucia, Australia (S 27° 29.9495' E 153° 0.0025'). A total of six plants with undefined-sex were selected for leaf-tissue collection. The leaf-tissue samples were excised and immediately snap-frozen in liquid nitrogen, then stored in − 80 °C until RNA isolation. All frozen samples were individually pulverised using a Retsch TissueLyser (Retsch, Haan, Germany) at a frequency of 30/S for 1 min. Approximately one gram of pulverized leaf-tissue powder per sample was used for RNA isolation.

### RNA isolation and quality control

Total RNAs from all leaf-tissue samples were successfully extracted using a two-step protocol. This protocol included a CTAB method followed by the use of a Qiagen RNeasy Plant mini kit (#74134, Qiagen, Valencia, CA, United States) to assure the complete removal of contaminating genomic DNA [63]. The quality, integrity, and quantity of isolated RNA samples were evaluated using a NanoDrop8000 spectrophotometer (ThermoFisher Scientific, Wilmington, DE, USA) for the initial screen and a 2100 Agilent Bioanalyzer (Agilent Technologies, Santa Clara, CA, USA).

### Transcriptome sequencing and data analysis

#### PacBio Iso-Seq library preparation

Total RNAs from all leaf-tissue samples were mixed and sequenced following the PacBio Iso-Seq Protocol. For the preparation of the Iso-Seq library, the NEBNext Single Cell/Low Input cDNA Synthesis, Amplification Module (NEB, E6421S), and the SMRTbell Express Template Prep Kit 2.0 (PacBio, 100-938-900) were used based on the standard protocol for the Iso-Seq Express Template Preparation for Sequel and Sequel II systems (PacBio, Part # 101-763-800 v1). A total of 300 ng of RNA per sample was reversely transcribed into cDNA and amplified to produce a minimum cDNA quantity of 160 ng for library preparation. The amplified cDNA was filtered with ProNex beads (Promega, NG2001) following the Iso-Seq protocol into two libraries using two different workflows: standard and long transcripts workflows. The two library workflows differ based on the purification beads ratio. The standard workflow requires 86 μL of ProNex beads, whereas 82 μL is needed to conduct the long workflow filtration. Additionally, a second round of amplification and purification is necessary for long transcripts workflow preparation [64]. The additional steps required by the long workflow is expected to enrich the dataset for longer isoforms transcript (3 × 10^3^ kb >) comparing to an enrichment aim of standard isoforms transcript length of 2 × 10^3^ Kb. Filtered and size-selected cDNA (160–500 ng) was used in a DNA damage repair reaction, followed by an end-repair/A-tailing reaction. Overhang adapters were ligated to the A-tailed library fragments, followed by ProNex beads filtration. The fragments from both standard and long libraries were quantified on the Qubit fluorometer using the Qubit dsDNA HS assay kit (Invitrogen, Q32854), and evaluated on the Agilent BioAnalyzer 2100 using the High Sensitivity DNA kit (Agilent, 5067–4626).

#### PacBio sequel II sequencing

The two libraries were prepared for sequencing following the standard protocol for diffusion loading with ProNex bead filtration, using Sequencing Primer v.4, the Sequel II DNA Internal Control v.1, and Sequel II Binding Kit v.2.0 and 2.1 for both long and standard transcript workflow libraries, respectively. Sequencing was performed using the PacBio Sequel II (software/chemistry v.8.0.0). Each polymerase-bound library was sequenced with one SMRT cell, a 24-h movie time and a two-h pre-extension using the Sequel II Sequencing 2.0 Kit (PacBio, 101-820-200) and SMRT Cell 8 M (PacBio, 101-389-001).

### The removal of isoforms redundancy (Cd-Hit)

The high-quality HQ isoforms post Cd-Hit analysis with 99% sequence identity threshold was preformed to remove the merged dataset of reference Iso-Seq two libraries redundancy using OmicsBox software (v.2.0) with default parameters (band length: 20 and length cut-off: 10) [65–67].

### Data analyses of Iso-Seq and consensus circular sequence (CCS)

The CCS application (v.4.0.0) in SMRT Link (v.8.0.0) was used with default settings of to produce CCS reads. The CCS reads were used as input for Iso-Seq analysis with the default settings of the Iso-Seq3 application (v.3.2.2) in SMRT Link (v.8.0.0) (Additional file 1: Table S5 and S6). The CCS raw sequences for both standard and long workflows were submitted to NCBI website under the following library IDs: CCS Standard workflow (SW) and CCS Long workflow (LW), respectively.

### Transcriptome completeness analysis

The completeness of the jojoba Iso-Seq reference transcriptome was evaluated using the 37,680 genes obtained from the jojoba male genome [15]. BUSCO v.4.1.2 as also used to assess the transcriptome completeness using default parameters (BLAST e-value (1.0E-10), mode (transcriptome), Linage (Plants)). The jojoba Iso-Seq transcriptome was aligned against 430 conserved single-copy orthologs in the Viridiplantae_odb10 BUSCO database.

### Functional annotation

Gene names were determined for the isoforms using Basic Local Alignment Search Tool (BLAST) with an e-value threshold of 1e-10, 10 BLAST hits, and 33 HSP length cut-off in OmicsBox v.2.0. Gene functions were assigned to the isoform using Gene Ontology (GO) terms associated with the BLAST result. InterProScan database was also used to enrich the annotations with GO terms based on sequence motifs/domains. GO terms not relevant to plants were removed using the GO-Slim tool. The Kyoto Encyclopedia of Genes and Genomes (KEGG) pathway enrichment analysis was generated using OmicsBox v.2.0 for all isoforms from the jojoba reference Iso-Seq transcriptome.

### Isoform characterisation

The isoforms of jojoba reference Iso-Seq transcriptome were categorised into eight structural classifications based on SQANTI3 (v.4.3, https://github.com/ConesaLab/SQANTI3) using jojoba reference genome and annotation [15, 68].

### The prediction of coding potential regions

The built-in Coding-Potential Assessment Tool (CPAT) software in OmicsBox v.2.0 [69] was used to assess the coding potential of isoform transcripts in the protein databases with default setting and *Arabidopsis thaliana* as a prebuilt model [25]. The predictions of the ORFs were conducted using the package of TransDecoder v.5.5.0 (https://github.com/sghignone/TransDecoder) with default settings [70]. The ORFs per transcript were selected with peptides length greater than or equal 100 aa. The longest ORFs from the overlapped frames were isolated by TransDecoder. The candidate coding potential regions were searched against the Pfam protein database v.32, using HMMER v.3.2.1.

### Supplementary Information

**Additional file 1: Figure S1**. Distribution of unique transcript isoforms from the long workflow across genes with CDS length between one to three thousand base pairs. Common transcript isoforms derived from SW and LW mapped to each of the CDS sequences are shown as blue bars and had a percentage of 36.3%, the unique transcripts isoforms derived from the SW are shown as black bars and had a percentage of 26.6%, while the unique transcripts isoforms derived from the LW and shown as orange bars had a percentage of 37.1%. Across all the isoforms, the average ratio of unique isoforms generated by LW / total isoforms by the SW generated isoforms was 0.65. Transcript isoforms shown here are non-redundant CD-Hit CCS sequences at 99% similarity. Mapping was undertaken using the “enable long-read spliced alignment” option in Minimap2 and executed via the CLC Genomics Workbench. **Figure S2**. Distribution of unique transcript isoforms from the long workflow across genes with CDS length between three to five thousand base pairs. Common transcript isoforms derived from SW and LW mapped to each of the CDS sequences are shown as blue bars and had a percentage of 26.9%, the unique transcripts isoforms derived from the SW are shown as black bars and had a percentage of 25.2%, while the unique transcripts isoforms derived from the LW and shown as orange bars had a percentage of 47.9%. Across all the isoforms, the average ratio of unique isoforms generated by LW / total isoforms by the SW generated isoforms was 1.0. Transcript isoforms shown here are non-redundant Cd-Hit CCS sequences at 99% similarity. Mapping was undertaken using the “enable long-read spliced alignment” option in Minimap2 and executed via the CLC Genomics Workbench. **Figure S3**. BUSCO v.5.1.2 analysis of four different datasets (long, standard, merged, and non-redundant Cd-Hit) for jojoba (Simmondsia chinensis) transcriptome Iso-Seq reference using the viridiplantae_odb10 dataset. The x-axis describes the percentage of complete and single copy, complete and duplicated, fragmented and missing BUSCO and the y-axis show. **Figure S4**. Number of transcript isoforms aligned to the InterProScan (IPS) families during the jojoba (Simmondsia chinensis) annotation. **Figure S5**. Gene Ontology (GO) classification of the jojoba (Simmondsia chinensis) Iso-Seq transcriptome reference (A) biological process, (B) molecular function, (C) cellular component. **Figure S6**. The top 11 KEGG pathways enrichment in the jojoba (Simmondsia chinensis) transcriptome Iso-Seq transcripts reference. **Figure S7**. Coding potential sequences for the non-redundant (Cd-Hit) jojoba (Simmondsia chinensis) transcriptome Iso-Seq reference including the four categories complete, 5’ potential, 3’ potential, and internal. **Figure S8**. Coding potential sequences for the novel isoforms in the jojoba (Simmondsia chinensis) transcriptome Iso-Seq reference including the four categories complete, 5’ potential, 3’ potential, and internal. **Table S4**. BUSCO analysis of four different datasets (long, standard, merged, and non-redundant Cd-Hit) for jojoba (Simmondsia chinensis) transcriptome Iso-Seq reference using the viridiplantae_odb10 dataset. **Table S5**. Default settings of consensus circular sequence (CCS) analysis used for jojoba (Simmondsia chinensis) Iso-Seq sequencing process. **Table S6**. Default settings of isoform sequencing (Iso-Seq) analysis used for jojoba (Simmondsia chinensis) water-stress RNA transcript isoforms. **Table S7**. Sequence coverage percentage for isoforms aligned to four jojoba (Simmondsia chinensis) apetala genes sequence.

**Additional file 2: Table S1**. Isoforms related to all jojoba (Simmondsia chinensis) genes with length range that falls between 1,000 and 3,000 base pair (bp) including library workflow type, uniqueness to long workflow library, ratio of long isoforms to standard workflow library, and all genes’ CDSs length.

**Additional file 3: Table S2**. Isoforms related to all jojoba (Simmondsia chinensis) genes with length range that falls between 3,000 and 5,000 base pair (bp) including dataset type, uniqueness to long workflow library, ratio of long isoforms to standard workflow library, and all genes CDS length.

**Additional file 4: Table S3**. Isoforms related to all jojoba (Simmondsia chinensis) genes with length over 5,000 base pair (bp) including workflow library type, uniqueness to long library workflow, ratio of long isoforms to standard workflow library, and all genes CDS length.

## Data Availability

Jojoba long-read reference Iso-Seq transcriptome data is available as Biosample ID: SAMN30551629 (https://www.ncbi.nlm.nih.gov/sra?LinkName=biosample_sra&from_uid=30551629), at the NCBI website under the BioProject ID: PRJNA694450 (https://www.ncbi.nlm.nih.gov/bioproject/PRJNA694450).

## Abbreviation

aa: Amino acid
Apt2: Apetala2
AS: Alternative splicing
ASEs: Alternative splicing events
BLAST: Basic local alignment search tool
bp: Base pair
BP: Biological process
BUSCO: Benchmarking Universal Single-Copy Orthologs
CC: Cellular component
CCSs: Circular consensus sequences
Cd-Hit: Cluster Database at High Identity with Tolerance
CDPK: Calcium-dependent protein kinases
CDS: Coding sequence
CPAT: Coding Potential Assessment Tool
DEA: Differentially expressed
DNA: Deoxyribonucleic acid
FSM: Full splice junction match
GOs: Gene ontologies
GTFs: General transcription factors
HQ isoforms: High-quality isoforms
HQ: High quality
ILR3: IAA-leucine resistant3
IPS: InterProScan
IR: Intron retention
ISM: Incomplete splice match
Iso-Seq: Isoforms sequencing
kb: Kilo base pair
KEGG: Kyoto Encyclopedia of Genes and Genomes
LW: Long workflow
MAPQ: Mapping quality
MED23: Mediator23
MF: Molecular function
Min: Minute
ncRNAs: Non-coding RNAs
NIC: Novel in catalog
NMD: Nonsense-mediated mRNA decay
NNIC: Novel not in catalog
NR: Non-redundant plant protein sequences
NTP: Nucleoside triphosphate
ORFs: Open reading frames
P-loop NTH: Phosphate-loop containing nucleoside triphosphate hydrolase
RNA: Ribonucleic acid
ROS: Reactive oxygen species
SW: Standard workflow
TPH: Tetratricopeptide-like helical
