# Supplementary material for: Long read sequencing to reveal the full complexity of a plant transcriptome by targeting both standard and long workflows
Source: Plant Methods. 2023 Oct 21;19:112. doi: 10.1186/s13007-023-01091-1 (PMC10589961; doi:10.1186/s13007-023-01091-1)
Supplement: Supplementary file 1 — Additional file 1: Figure S1. Distribution of unique transcript isoforms from the long workflow across genes with CDS length between one to three thousand base pairs. Common transcript isoforms derived from SW and LW mapped to each of the CDS sequences are shown as blue bars and had a percentage of 36.3%, the unique transcripts isoforms derived from the SW are shown as black bars and had a percentage of 26.6%, while the unique transcripts isoforms derived from the LW and shown as orange bars had a percentage of 37.1%. Across all the isoforms, the average ratio of unique isoforms generated by LW / total isoforms by the SW generated isoforms was 0.65. Transcript isoforms shown here are non-redundant CD-Hit CCS sequences at 99% similarity. Mapping was undertaken using the “enable long-read spliced alignment” option in Minimap2 and executed via the CLC Genomics Workbench. Figure S2. Distribution of unique transcript isoforms from the long workflow across genes with CDS length between three to five thousand base pairs. Common transcript isoforms derived from SW and LW mapped to each of the CDS sequences are shown as blue bars and had a percentage of 26.9%, the unique transcripts isoforms derived from the SW are shown as black bars and had a percentage of 25.2%, while the unique transcripts isoforms derived from the LW and shown as orange bars had a percentage of 47.9%. Across all the isoforms, the average ratio of unique isoforms generated by LW / total isoforms by the SW generated isoforms was 1.0. Transcript isoforms shown here are non-redundant Cd-Hit CCS sequences at 99% similarity. Mapping was undertaken using the “enable long-read spliced alignment” option in Minimap2 and executed via the CLC Genomics Workbench. Figure S3. BUSCO v.5.1.2 analysis of four different datasets (long, standard, merged, and non-redundant Cd-Hit) for jojoba (Simmondsia chinensis) transcriptome Iso-Seq reference using the viridiplantae_odb10 dataset. The x-axis describes the percentage of c [file 13007_2023_1091_MOESM1_ESM.docx]

**Supplementary figures and tables:**

***Figure S1*.** Distribution of unique transcript isoforms from the long workflow across genes with CDS length between one to three thousand base pairs.

Common transcript isoforms derived from SW and LW mapped to each of the CDS sequences are shown as blue bars and had a percentage of 36.3%, the unique transcripts isoforms derived from the SW are shown as black bars and had a percentage of 26.6%, while the unique transcripts isoforms derived from the LW and shown as orange bars had a percentage of 37.1%. Across all the isoforms, the average ratio of unique isoforms generated by LW / total isoforms by the SW generated isoforms was 0.65. Transcript isoforms shown here are non-redundant CD-Hit CCS sequences at 99% similarity. Mapping was undertaken using the “enable long-read spliced alignment” option in Minimap2 and executed via the CLC Genomics Workbench.

***Figure S2*.** Distribution of unique transcript isoforms from the long workflow across genes with CDS length between three to five thousand base pairs.

Common transcript isoforms derived from SW and LW mapped to each of the CDS sequences are shown as blue bars and had a percentage of 26.9%, the unique transcripts isoforms derived from the SW are shown as black bars and had a percentage of 25.2%, while the unique transcripts isoforms derived from the LW and shown as orange bars had a percentage of 47.9%. Across all the isoforms, the average ratio of unique isoforms generated by LW / total isoforms by the SW generated isoforms was 1.0. Transcript isoforms shown here are non-redundant Cd-Hit CCS sequences at 99% similarity. Mapping was undertaken using the “enable long-read spliced alignment” option in Minimap2 and executed via the CLC Genomics Workbench.

***Figure S3*.** BUSCO v.5.1.2 analysis of four different datasets (long, standard, merged, and non-redundant Cd-Hit) for jojoba (*Simmondsia chinensis*) transcriptome Iso-Seq reference using the viridiplantae_odb10 dataset. The x-axis describes the percentage of complete and single copy, complete and duplicated, fragmented and missing BUSCO and the y-axis show.

***Figure S4*.** Number of transcript isoforms aligned to the InterProScan (IPS) families during the jojoba (*Simmondsia chinensis*) annotation.

***Figure S5*.** Gene Ontology (GO) classification of the jojoba (*Simmondsia chinensis*) Iso-Seq transcriptome reference (A) biological process, (B) molecular function, (C) cellular component.

***Figure S6.*** The top 11 KEGG pathways enrichment in the jojoba (*Simmondsia chinensis*) transcriptome Iso-Seq transcripts reference.

***Figure S7*.** Coding potential sequences for the non-redundant (Cd-Hit) jojoba (*Simmondsia chinensis*) transcriptome Iso-Seq reference including the four categories complete, 5’ potential, 3’ potential, and internal.

***Figure S8*.** Coding potential sequences for the novel isoforms in the jojoba *(Simmondsia chinensis)* transcriptome Iso-Seq reference including the four categories complete, 5’ potential, 3’ potential, and internal.

***Table S1.*** Isoforms related to all jojoba (*Simmondsia chinensis*) genes with length range that falls between 1,000 and 3,000 base pair (bp) including library workflow type, uniqueness to long workflow library, ratio of long isoforms to standard workflow library, and all genes’ CDSs length.

***Table S2.*** Isoforms related to all jojoba (*Simmondsia chinensis*) genes with length range that falls between 3,000 and 5,000 base pair (bp) including dataset type, uniqueness to long workflow library, ratio of long isoforms to standard workflow library, and all genes CDS length.

***Table S3.*** Isoforms related to all jojoba (*Simmondsia chinensis*) genes with length over 5,000 base pair (bp) including workflow library type, uniqueness to long library workflow, ratio of long isoforms to standard workflow library, and CDS length of all genes.

***Table S4*.** BUSCO analysis of four different datasets (long, standard, merged, and non-redundant Cd-Hit) for jojoba (*Simmondsia chinensis*) transcriptome Iso-Seq reference using the viridiplantae_odb10 dataset.

***Table S5*.** Default settings of consensus circular sequence (CCS) analysis used for jojoba (*Simmondsia chinensis*) Iso-Seq sequencing process.

***Table S6*.** Default settings of isoform sequencing (Iso-Seq) analysis used for jojoba (*Simmondsia chinensis)* water-stress RNA transcript isoforms.

***Table S7.*** Sequence coverage percentage for isoforms aligned to four jojoba (*Simmondsia chinensis*) apetala genes sequence.


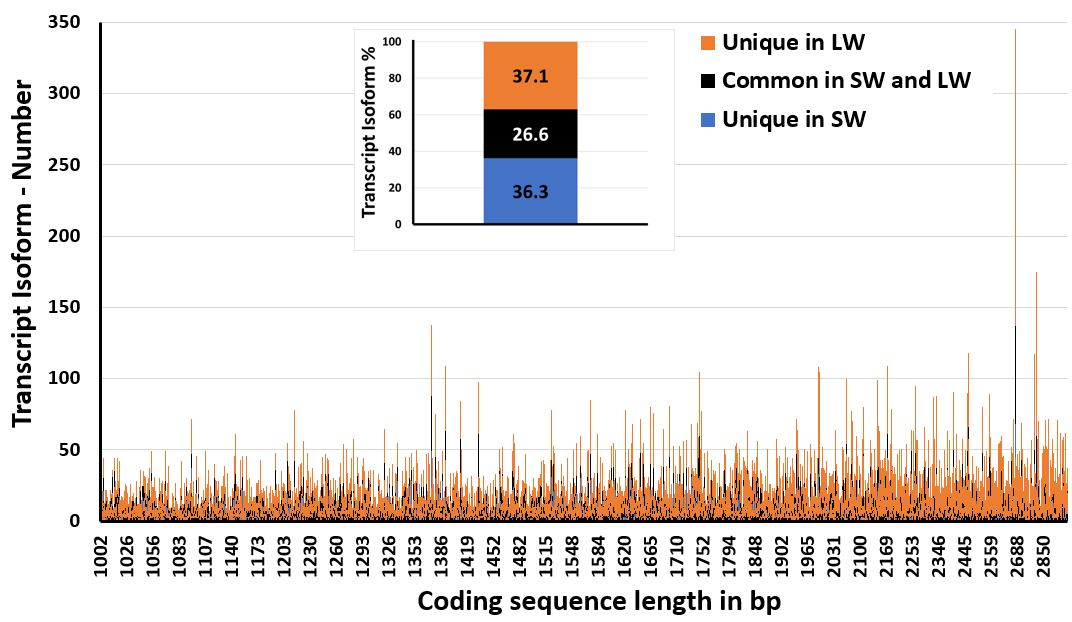
*Figure S1.* Distribution of unique transcript isoforms from the long workflow across genes with CDS length between one to three thousand base pairs.

Common transcript isoforms derived from SW and LW mapped to each of the CDS sequences are shown as blue bars and had a percentage of 36.3%, the unique transcripts isoforms derived from the SW are shown as black bars and had a percentage of 26.6%, while the unique transcripts isoforms derived from the LW and shown as orange bars had a percentage of 37.1%. Across all the isoforms, the average ratio of unique isoforms generated by LW / total isoforms by the SW generated isoforms was 0.65. Transcript isoforms shown here are Cd-Hit CCS sequences at 99% similarity. Mapping was undertaken using the “enable long-read spliced alignment” option in Minimap2 and executed via the CLC Genomics Workbench.


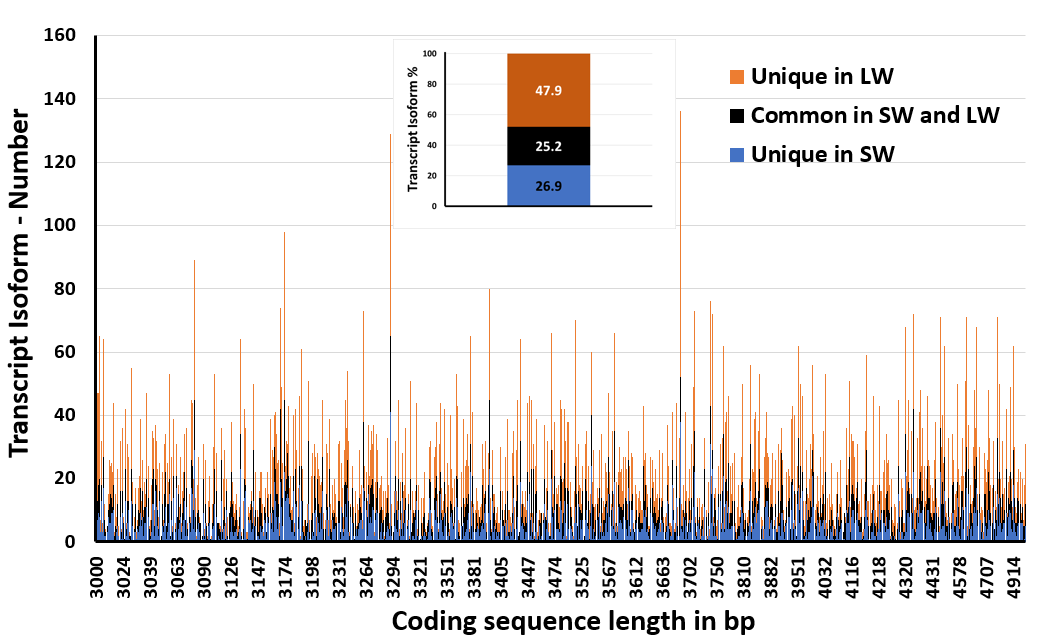


*Figure S2.* Distribution of unique transcript isoforms from the long workflow across genes with CDS length between three to five thousand base pairs.

Common transcript isoforms derived from SW and LW mapped to each of the CDS sequences are shown as blue bars and had a percentage of 26.9%, the unique transcripts isoforms derived from the SW are shown as black bars and had a percentage of 25.2%, while the unique transcripts isoforms derived from the LW and shown as orange bars had a percentage of 47.9%. Across all the isoforms, the average ratio of unique isoforms generated by LW / total isoforms by the SW generated isoforms was 1.0. Transcript isoforms shown here are CD-Hit CCS sequences at 99% similarity. Mapping was undertaken using the “enable long-read spliced alignment” option in Minimap2 and executed via the CLC Genomics Workbench.

*Figure S3.* BUSCO v.5.1.2 analysis of four different dataset (long, standard, merged, and non-redundant Cd-Hit) for jojoba *(Simmondsia chinensis)* transcriptome Iso-Seq reference using the viridiplantae_odb10 dataset. The x-axis describes the percentage of complete and single copy, complete and duplicated, fragmented and missing BUSCO and the y-axis show.

*Figure S4.* Number of transcript isoforms aligned to the InterProScan (IPS) families during the *jojoba (Simmondsia chinensis)* annotation.

*Figure S5.* Gene Ontology (GO) classification of the jojoba *(Simmondsia chinensis)* Iso-Seq transcriptome reference (A) biological process, (B) molecular function, (C) cellular component.

Figure S6. The top 11 KEGG pathways enrichment in the jojoba (Simmondsia chinensis) transcriptome Iso-Seq transcripts reference.

Figure S7. Coding potential sequences for the Cd-Hit jojoba (Simmondsia chinensis) transcriptome Iso-Seq reference including the four categories complete, 5’ potential, 3’ potential, and internal.

Figure S8. Coding potential sequences for the novel isoforms in the jojoba (Simmondsia chinensis) transcriptome Iso-Seq reference including the four categories complete, 5’ potential, 3’ potential, and internal.

*Table S1.* Isoforms related to all jojoba *(Simmondsia chinensis)* genes with length range that falls between 1,000 and 3,000 base pair (bp) including library workflow type, uniqueness to long workflow library, ratio of long isoforms to standard workflow library, and all genes’ CDSs length.

(Excel file of Table S1 is included)

*Table S2.* Isoforms related to all jojoba *(Simmondsia chinensis)* genes with length range that falls between 3,000 and 5,000 base pair (bp) including dataset type, uniqueness to long workflow library, ratio of long isoforms to standard workflow library, and all genes’ CDSs length.

(Excel file of Table S2 is included)

*Table S3.* Isoforms related to all jojoba *(Simmondsia chinensis)* genes with length over 5,000 base pair (bp) including workflow library type, uniqueness to long library workflow, ratio of long isoforms to standard workflow library, and all genes’ CDSs length.

(Excel file of Table S3 is included)

*Table S4*. BUSCO analysis of four different datasets (long, standard, merged, and non-redundant (Cd-Hit) for jojoba (*Simmondsia chinensis*) transcriptome Iso-Seq reference using the viridiplantae_odb10 dataset.

|  | Total complete (%) | Single (%) | Duplicated (%) | Fragmented (%) | Missing (%) | Total core genes# |
| --- | --- | --- | --- | --- | --- | --- |
| long | (375) 88.2 | (83) 19.5 | (292) 68.7 | (14) 3.7 | (36) 8.5 | 425 |
| Standard | (409) 96.2 | (34) 8.0 | (375) 88.2 | (4) 0.9 | (12) 2.8 |  |
| Merged | (412) 96.9 | (17) 4.0 | (395) 92.9 | (3) 0.7 | (10) 2.4 |  |
| Cd-Hit | (412) 96.9 | (38) 8.9 | (374) 88.0 | (3) 0.7 | (10) 2.4 |  |

*Table S5.* Default setting of consensus circular sequence (CCS) analysis used for jojoba *(Simmondsia chinensis)* Iso-Seq sequencing process.

| Parameter | Default value |
| --- | --- |
| Minimum number of passes | 3 |
| Minimum predicted accuracy | 0.99 |
| Minimum CCS read length | 10 |
| Maximum CCS read length | 50000 |

*Table S6. Default setting of isoform sequencing (Iso-Seq) analysis used for jojoba (Simmondsia chinensis) water-stress RNA transcript isoforms.*

| Parameter | Default value |
| --- | --- |
| Run clustering | On (generates consensus isoforms) |
| Minimum mapped length (bp) | 50 |
| Maximum fuzzy junction difference (bp) | 5 |
| Minimum mapped concordance (%) | 95 |
| Minimum passes for high quality isoforms | 7 |
| Minimum mapped coverage (%) | 99 |
| Require and trim Poly(A) tail | On |

*Table S7. Sequence coverage percentage for isoforms aligned to four Jojoba (Simmondsia chinensis) apetala genes sequence.*

| Apetala genes | Gene size (bp) | Isoform ID | Isoform size (bp) | Coverage (%) | Average coverage (%) |
| --- | --- | --- | --- | --- | --- |
| **Apetala2-1** | 12,538 | Multiple_HQ_transcript/171107 | 2,824 | 23 | 18 |
|  |  | Multiple_HQ_transcript/105509 | 3,316 | 26 |  |
|  |  | Multiple_HQ_transcript/412095 | 643 | 5 |  |
|  |  | Multiple_HQ_transcript/327430 | 1,787 | 14 |  |
|  |  | Multiple_HQ_transcript/315531 | 1,880 | 15 |  |
|  |  | Multiple_HQ_transcript/222064 | 2,503 | 20 |  |
|  |  | Multiple_HQ_transcript/366229 | 1,420 | 11 |  |
|  |  | Multiple_HQ_transcript/270607 | 2,192 | 17 |  |
|  |  | Multiple_HQ_transcript/100081 | 3,363 | 27 |  |
|  |  | Multiple_HQ_transcript/111394 | 3,253 | 26 |  |
| **Apetala2-2** | 5,823 | Multiple_HQ_transcript/303053 | 1,970 | 34 | 43 |
|  |  | Multiple_HQ_transcript/257514 | 2,274 | 39 |  |
|  |  | Multiple_HQ_transcript/105872 | 3,312 | 57 |  |
| **Apetala2-3** | 8,881 | Multiple_HQ_transcript/265232 | 2,225 | 25 | 21 |
|  |  | Multiple_HQ_transcript/264460 | 2,218 | 25 |  |
|  |  | Multiple_HQ_transcript/253270 | 2,302 | 26 |  |
|  |  | Multiple_HQ_transcript/409273 | 705 | 8 |  |
|  |  | Multiple_HQ_transcript/319944 | 1,834 | 21 |  |
| **Apetala2-4** | 7,590 | Multiple_HQ_transcript/361458 | 1,476 | 19 | 25 |
|  |  | Multiple_HQ_transcript/358337 | 1,508 | 20 |  |
|  |  | Multiple_HQ_transcript/382288 | 1,207 | 16 |  |
|  |  | Multiple_HQ_transcript/303247 | 1,969 | 26 |  |
|  |  | Multiple_HQ_transcript/295283 | 2,026 | 27 |  |
|  |  | Multiple_HQ_transcript/297282 | 1,976 | 26 |  |
|  |  | Multiple_HQ_transcript/293887 | 2,035 | 27 |  |
|  |  | Multiple_HQ_transcript/320398 | 1,844 | 24 |  |
|  |  | Multiple_HQ_transcript/99492 | 3,371 | 44 |  |
|  |  | Multiple_HQ_transcript/321893 | 1,832 | 24 |  |
